# Temporal and Spatial Patterns of Inflammation and Tissue Injury in Patients with Postoperative Respiratory Failure after Lung Resection Surgery: A Nested Case–Control Study

**DOI:** 10.3390/ijms241210051

**Published:** 2023-06-13

**Authors:** Jay Kormish, Tejas Ghuman, Richard Y. Liu, Sadeesh K. Srinathan, Lawrence Tan, Kristen Graham, Stephanie Enns, Gordon Buduhan, Andrew J. Halayko, Christopher D. Pascoe, Biniam Kidane

**Affiliations:** 1 Section of Thoracic Surgery, Department of Surgery, Health Sciences Centre, Winnipeg, MB R3A 1R9, Canada; jay.kormish@umanitoba.ca (J.K.);; 2 Department of Surgery, Rady Faculty of Health Sciences, Max Rady College of Medicine, University of Manitoba, Winnipeg, MB R3A 1R9, Canada; 3 Children’s Hospital Research Institute of Manitoba, Winnipeg, MB R3A 1R9, Canada; 4 Department of Physiology and Pathophysiology, Rady Faculty of Health Sciences, Max Rady College of Medicine, University of Manitoba, Winnipeg, MB R3A 1R9, Canada

**Keywords:** one-lung ventilation (OLV), acute lung injury (ALI), ventilation-induced lung injury (VILI), acute respiratory distress syndrome (ARDS), postoperative pulmonary complications (PPCs)

## Abstract

Thoracic surgeries involving resection of lung tissue pose a risk of severe postoperative pulmonary complications, including acute respiratory distress syndrome (ARDS) and respiratory failure. Lung resections require one-lung ventilation (OLV) and, thus, are at higher risk of ventilator-induced lung injury (VILI) attributable to barotrauma and volutrauma in the one ventilated lung, as well as hypoxemia and reperfusion injury on the operated lung. Further, we also aimed to assess the differences in localized and systemic markers of tissue injury/inflammation in those who developed respiratory failure after lung surgery versus matched controls who did not develop respiratory failure. We aimed to assess the different inflammatory/injury marker patterns induced in the operated and ventilated lung and how this compared to the systemic circulating inflammatory/injury marker pattern. A case–control study nested within a prospective cohort study was performed. Patients with postoperative respiratory failure after lung surgery (*n* = 5) were matched with control patients (*n* = 6) who did not develop postoperative respiratory failure. Biospecimens (arterial plasma, bronchoalveolar lavage separately from ventilated and operated lungs) were obtained from patients undergoing lung surgery at two timepoints: (1) just prior to initiation of OLV and (2) after lung resection was completed and OLV stopped. Multiplex electrochemiluminescent immunoassays were performed for these biospecimen. We quantified 50 protein biomarkers of inflammation and tissue injury and identified significant differences between those who did and did not develop postoperative respiratory failure. The three biospecimen types also display unique biomarker patterns.

## 1. Introduction

Lung cancers are the leading global cause of cancer-related deaths [1]. Surgical resection of lung cancers is the standard of care for curative treatment [2], but surgical manipulation of the operated lung combined with mechanical ventilation of the contralateral lung can increase the risk of acute lung injury (ALI) and ventilation-induced lung injury (VILI) and the occurrence of serious postoperative pulmonary complications (PPCs), including respiratory failure and acute respiratory distress syndrome (ARDS) [3].

All surgeries performed under general anesthesia require artificial ventilation using a mechanical ventilator [4,5,6,7]. Normal ventilation in an awake person is achieved by creating negative pressure that *pulls* a volume of air into the lungs. However, mechanical ventilation uses positive pressure to *push* air into the lungs through an endotracheal tube (ETT). Patients undergoing lung surgery are at risk for lung damage/injury for two major reasons [8,9,10]. Firstly, the lung being operated on must be deflated so that surgeons can work on it; therefore, unlike for surgeries on other organs, all mechanical ventilation is delivered to only one lung, the one opposite the site of surgery [11]. Known as one-lung ventilation (OLV), this increases the potential for damage to the single ventilated lung [9,10,11]. Secondly, most patients who have lung surgery have abnormal lungs, making them more vulnerable to lung damage with mechanical ventilation, increasing the risk for developing respiratory complications [4,5,6,7,11]. Forcing air into the lungs has significant potential to cause injury if the frequency, pressure, and volume are not carefully controlled. As such, mechanical ventilation represents a major risk factor for post-operative respiratory complications; these include failure to return to normal oxygen levels, pneumonia, and ALI, which contribute to respiratory failure, which can in turn lead to death. Patients may also develop compromised breathing capacity that requires them to become permanently dependent on supplemental home oxygen; this results in a significant loss of quality of life. In addition to ventilation-induced trauma, patients undergoing lung surgery are also subjected to mechanical surgical trauma to the lung that is being operated on. For example, a patient having a right upper lobe removal will be subjected to physical trauma to the remaining right middle and lower lobes due to the tissue handling and retraction that are required for the removal of the upper lobe. This surgical trauma may also induce changes to both local lung and systemic environments, which may increase the risk of respiratory complications.

ALI following lung resection has an estimated incidence of 2.45%; however, surgeries requiring more extensive tissue removal have an increased incidence of ALI, with ALI occurring in 7.9% of pneumonectomies (which is the removal of the entire lung on one side of the body) [3,12]. Respiratory failure as a result of ALI is the major cause of mortality following thoracic surgery (mortality rates ranging from 25 to 45%) [3,5,12,13,14,15]. The etiology and pathogenesis of postoperative ALI is not fully understood, and the mechanism is expected to be complex and multifactorial [16]. A multiple insult model has been developed in animal models to account for the incidence and variability of ALI [17,18]. The contributing factors to ALI after lung surgery are multifactorial; there are factors that can be attributed to OLV (i.e., VILI) and those that can be attributed to surgical trauma (i.e., physical trauma of surgical manipulation, hypoxemia and reperfusion injuries induced by operated lung deflation/reinflation) [3,4,5,15,16].

Our best estimates of the risk of developing PPCs, including acute or chronic respiratory failure, after lung surgery are based on large-scale registry studies from both North American and Eurasian populations [19,20,21,22]. These studies identified key risk factors for the development of major pulmonary complications after lung surgery; these risk factors are unmodifiable, however, as these studies were focused on preoperative and basic surgical variables. These studies were used to derive predictive risk scores for the development of PPCs. Despite including registry data on 82,000 and 27,000 patients, respectively, these predictive scores never exceed an area under the curve (AUC) of 0.74 y in the accuracy of predicting cardiopulmonary complications and 30-day mortality after lung surgery [20]. The inclusion of all cardiopulmonary complications lumped together creates a limited applicability to respiratory failure and PPCs as specific outcomes. More importantly, prediction or accuracy is also limited by the absence of important risk factors, such as low diffusing capacity for carbon monoxide (DLCO), for example [23], as well as reliance on static clinical predictors of baseline states that are separated both temporally and causally from the pathogenetic pathway between surgery and the resultant respiratory failure.

In addition to operative variables such as the duration and complexity of surgery, exposure to mechanical ventilation alone may be a driver of ALI in patients undergoing major surgery. VILI can potentially occur through multiple mechanisms. It can induce mechanical stress [24], as well as oxidative damage [25]. During OLV, lung damage can result from lung overinflation (volutrauma), high inspiratory pressures (barotrauma), and oxidative stress resulting from hyperoxia (biotrauma) [5]. The operated lung is also likely to contribute to respiratory complications. The consequence of lung deflation and reinflation, pulmonary hypertension due to obligatory shunts, and reperfusion injury is yet to be defined in terms of risk assessment. These types of traumas are likely to be especially deleterious in patients undergoing lung resection for two major reasons. First, these operations typically use OLV; therefore, all the traumatic ventilatory forces described above are exerted on the one ventilated lung. Second, most patients who have lung surgery have abnormal lungs, making them more vulnerable to lung damage with mechanical ventilation, increasing the risk for developing PPCs. Chronic obstructive pulmonary disease (COPD) and/or interstitial lung disease (ILD) are chronic pulmonary diseases within the surgical population that make the ventilated lung more vulnerable to mechanical and inflammatory injury. The reduced respiratory function within these pathologies necessitate lung recruitment and increased oxygenation strategies, which further increases the risk of developing ALI. Although lung protective ventilation strategies have been proposed to minimize the trauma of OLV, there is significant variation among anesthesiologists in both the definition and implementation of these lung-protective strategies [9,10,11]. Most of this variation is due to a lack of a strong evidence base for lung-protective ventilation strategies for OLV surgeries.

Thus the effect of the preoperative patient condition and the combined insults of OLV and surgical resection contribute to creating a multiple-hit model, where the cumulative effects of many respiratory variables contribute to serious or long-term respiratory complications [5]. In this study, we aimed to assess and identify biomarker dynamics that are indicative of and may be predictors of postoperative respiratory failure after lung surgery. As our primary objective, we aimed to use a case–control method to assess the differences in localized and systemic markers of tissue injury and inflammation in those who developed respiratory failure after surgery versus matched controls who did not develop respiratory failure after surgery. As our secondary objective, we aimed to assess the different inflammatory and injury marker patterns induced in the operated and ventilated lung and how this compared to systemic circulating inflammatory and injury biomarker pattern. 

## 2. Results

### Biomarkers Associated with Respiratory Failure

**Figure 1:**
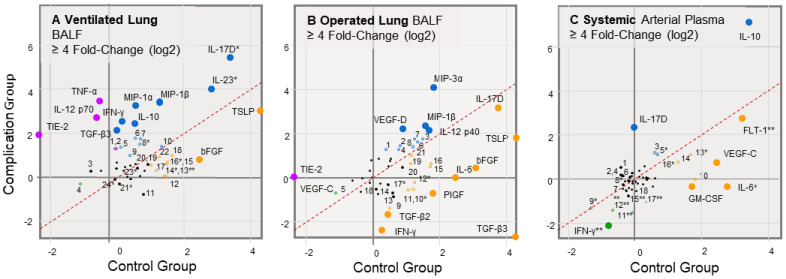
Fold-change comparisons for pre- to post-OLV biomarker mean concentrations within the complications patient group compared to the control patient group. Mean fold-change calculations were log2 transformed. Biomarker dynamics detected in the BALFs of the ventilated (**A**) and operated (**B**) lungs and arterial plasma (**C**). Biomarkers with greater than a 4-fold (large circle) and 2-fold (small open circle) change in post-OLV concentrations are indicated. Significance was analyzed using a two-way ANOVA with repeated measures for individual patient type or sample time and combined effects. (**A**) 1 IL-8, 2 IL-13, 3 IL-2, 4 IL-17C, 5 IL-4, 6 IL-1β, 7 IL-16, 8 IL-1α, 9 IL-5, 10 MIP-3α, 11 IL-21, 12 FLT-1, 13 IL-Ra, 14 IL-6, 15 IP-10, 16 TARC, 17 VEGF-D, 18 IL-12 p40/IL-23, 19 VEGF-A, 20 IL-15, 21 Eotaxin, 22 MCP-1, 23 IL-7, 24 TGF-β2. (**B**) 1 IL-17A, 2 IP-10, 3 TNF-α, 4 TARC, 5 IL-12 p70, 6 FLT-1, 7 MIP-1α, 8 IL-16, 9 IL-1β, 10 IL-17C, 11 IL-1α, 12 IL-22, 13 IL-2, 14 IL-8, 15 IL-10, 16 IL-5, 17 IL-21, 18 IL-Ra, 19 MCP-1, 20 VEGF-A, 21 TGF-β1. (**C**) 1 Eotaxin-3, 2 MIP-3α, 3 IL-17C, 4 IL-15, 5 IL-7, 6 MDC, 7 IP-10, 8 IL-12 p40, 9 IL-5, 10 IL-1Ra, 11 IL-1β, 12 VEGF-A, 13 IL-4, 14 IL-23, 15 TNF-α, 16 IL-27, 17 MCP-1, 18 Tie-2.

**Figure 2:**
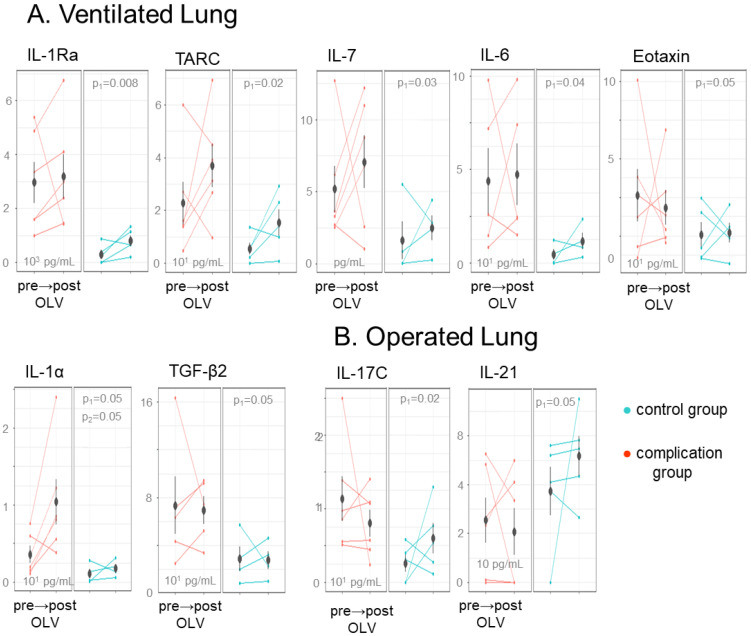
Biomarkers correlated with respiratory failure in the ventilated and operated lungs of OLV patients. Mean biomarker concentrations that differ between the complications and control patient groups within (**A**) the BALFs of the ventilated lung pre-OLV and post-OLV and (**B**) the BALFs of the operated lung pre-OLV to post-OLV. Biomarkers with a significant patient type effect (p_1_, α ≤ 0.05) are plotted with the mean group concentration and standard deviation indicated for each patient group.

**Figure 3:**
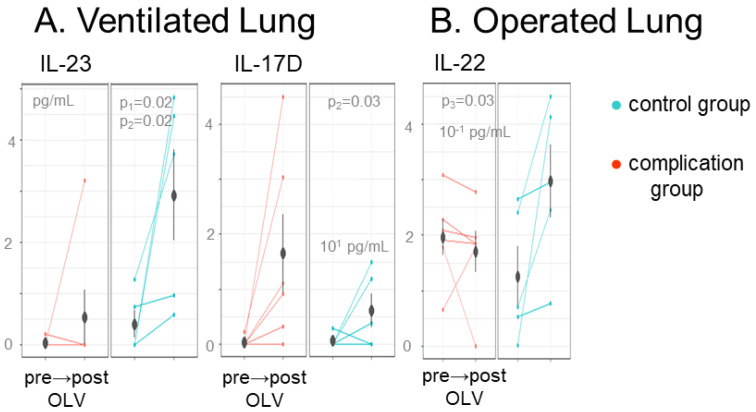
Biomarkers correlated with the OLV procedure in ventilated and operated lung of OLV patients. Mean biomarker concentrations that differ between the pre-OLV to post-OLV sampling within (**A**) the BALFs of the ventilated lung pre-OLV and post-OLV and (**B**) the BALFs of the operated lung pre-OLV to post-OLV. Biomarkers with a significant sample time effect (p_2_, α ≤ 0.05) or full effect (p_3_) are plotted with the mean group concentration and standard deviation indicated for each patient group.

**Figure 4:**
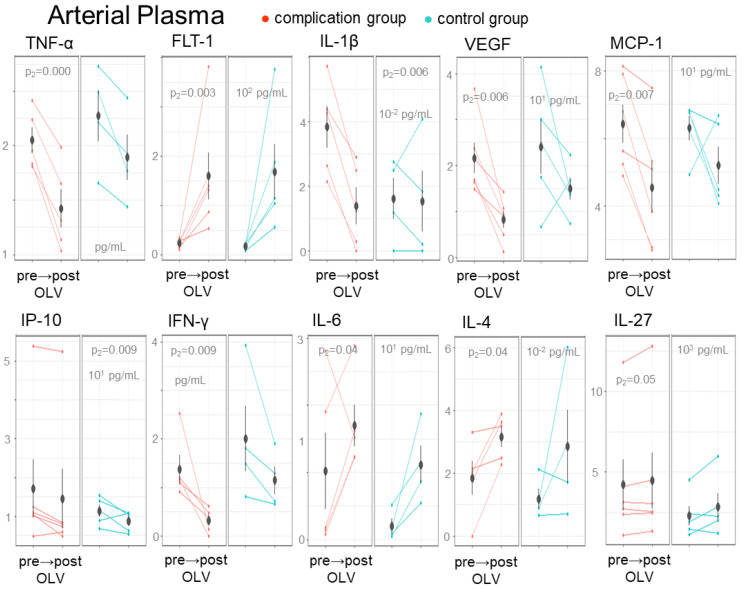
Biomarkers correlated with the OLV procedure in arterial plasma of OLV patients. Mean biomarker concentrations that differ between the pre-OLV to post-OLV sampling within the arterial plasma pre-OLV and post-OLV. Biomarkers with a significant sample time effect (p_2_, α ≤ 0.05) or full effect (p_3_, α ≤ 0.05) are plotted with the mean group concentration and standard deviation indicated for each patient group.

The bronchoalveolar lavage fluids (BALFs) from the ventilated compared to the operated lung display distinct biomarker dynamics in patients that have respiratory failure. The markers with an absolute or combined fold-change greater than four-fold are highlighted in fold-change plots for sample-specific and patient group comparisons (Figure 1). Changes in biomarker concentrations according to patient type (PT) and sample time (ST) were log_2_ transformed, ranked, and analyzed for the significance of interaction in a two-way ANOVA repeated measures analysis (Table 1 and Table 2). Biomarkers that met an inclusive α threshold α ≤ 0.05 for the patient type (PT, p_1_), sample time (ST, p_2_), and patient type:sample time (PT:ST, p_3_) full effect are plotted for visualization of dynamic trends (Figure 2, Figure 3 and Figure 4). Proinflammatory markers TNF-α, IL-12 (p70), IFN-γ, and IL-10 (Figure 1A,B) had a distinct increase in the patient cases with complications (i.e., those patients with postoperative respiratory failure), which was not observed in the control patients. INF-γ decreased in the arterial blood samples (p_2_ = 0.009; Figure 1C), and this dynamic was reflected in the operated lung (Figure 1B). IL-10 displayed an increasing trend in the ventilated lung of both patient groups (Figure 1A), but this increase was on average high in magnitude in the patients with complications. The elevated level of IL-10 in the arterial plasma (Figure 1C) was more similar to the dynamics in the ventilated lungs. An acute injury marker, IL-6, increased in the arterial plasma of controls (Figure 1C p_2_ = 0.04), but was unchanged in the complications group. This IL-6 dynamics was more closely reflected in the operated lung sampling (Figure 1B). Several chemotactic proteins MIP-1α and MIP-3α increased in the ventilated (Figure 1A) and the operated (Figure 1B) lungs, respectively, while MIP-1β had a similar increasing trend within both patient groups. Two biomarkers of innate or adaptive immunity IL-17D (sample time p_2_ = 0.03) and IL-23 (p_1_ = 0.02 and p_2_ = 0.02) exhibited a total fold-change of 4.0 and 2.2 within the ventilated lung, respectively. IL-17D displayed an increasing 5.2-fold trend within the arterial plasma of patients with complications and more closely reflected the ventilated lung dynamics. Several angiogenic biomarkers including the soluble FLT-1 receptor and the VEGF-C, VEGF-D, and PIGF growth factors displayed a complex pattern of expression in the operated lung and arterial plasma. Soluble FLT-1 increased 9.3- and 6.8-fold in the control and complications patient groups, respectively (p_2_ = 0.003). Two TGF-β isoforms may also have reciprocal patterns within the two lung samplings (Figure 1A,B) with TGF-β3 increasing in the ventilated lungs of the group with complications, while decreasing in both groups within the operated lungs.

Biomarker sampling was designed to determine if the operative lung and/or the ventilated lung (the lung subjected to OLV) contributed to the incidence and severity of postoperative respiratory complications. The majority of biomarkers identified in the two-way ANOVA repeated measures analysis as significant for patient-group-specific effects (p_1_, Figure 2A,B) including IL-1Ra (p_1_ = 0.008), TARC (p_1_ = 0.02), IL-7 (p_1_ = 0.03), IL-6 (p_1_ = 0.04), Eotaxin (p_1_ = 0.05), IL1α (p_1_ = 0.05), and TGF-β2 (p_1_ = 0.05) were detected to be higher.

Concentrations within the complications group within the ventilated lung sampling, but not in the operated lung. IL-17C (p_1_ = 0.02) was higher in the operated lung of complications patients, while IL-21 (p_1_ = 0.05) displayed the opposite effect with a lower concentration within the complications group (Figure 1B,C). Only three markers were significant for changes over sampling time (i.e., from pre- to post-OLV/surgery) (Figure 2A,B). IL-23 (p_2_ = 0.02) and IL-17D (p_2_ = 0.03) increased in both the complications and control groups in the ventilated lung sampling. IL-22 (p_2_ = 0.03) increased in the control group of the operated lung sampling, while this trend was deficient in the complications group.

The arterial plasma sampling pre-OLV to post-OLV (Figure 4) identified ten biomarkers with significant biomarker dynamics: TNF-α (p_2_ = 0.000), FLT-1 (p_2_ = 0.003), IL-1β (p_2_ = 0.006), VEGF (p_2_ = 0.006), MCP-1 (p_2_ = 0.007), IP-10 (p_2_ = 0.006), IFN-γ (p_2_ = 0.009), IL-6 (p_2_ = 0.04), IL-4 (p_2_ = 0.04), and IL-27 (p_2_ = 0.04). One biomarker IL-1β (p_3_ = 0.008) was identified as significant for the full patient type and sample time effect. The majority of the biomarkers including TNF-α (p_2_ = 0.000), VEGF (p_2_ = 0.006), MCP-1 (p_2_ = 0.007), IP-10 (p_2_ = 0.006), and IFN-γ (p_2_ = 0.009) decreased in the detection of systemic levels in both patient groups in response to OLV/surgery. This response was similar for IL-1β, but specific to the patients with complications. In contrast, FLT-1 (p_2_ = 0.003), IL-6 (p_2_ = 0.04), IL-4 (p_2_ = 0.04), and IL-27 (p_2_ = 0.04) displayed increasing concentrations in both patient groups. Only IL-6 was associated with patient-specific elevated levels with higher levels detected in the ventilated lungs of the complications group (Figure 2A).

## 3. Discussion

Our case–control study was able to detect biomarkers that differed significantly between the patient groups. In these analyses, biomarker levels in patients that presented postoperative respiratory complications (i.e., the cases) were compared to patients without postoperative respiratory complications (i.e., the controls). Within this matched study design, patients with similar preoperative variables were compared for differences in biomarker levels pre-OLV and for changes that occurred in response to OLV, herein defined as the sample time, including changes in biomarker concentration pre-OLV surgery vs. post-OLV surgery. The three sample types, ventilated lung BALFs, operated lung BALFs, and arterial plasma, displayed unique biomarker patterns. The ventilated lung demonstrated the greatest variability in biomarkers when compared to the operated lung. Only one biomarker, IL-6, had detectable dynamic patterns within two milieus including the ventilated lung and circulating systemic levels. The direct comparison of the fold-change dynamics between patient groups provided an informative composite of biomarker patterns, but trends were typically insignificant in the analysis. This may reflect the physiological range of individual biomarkers, the responsive properties of each biomarker, or the small sample size of the study, which lacked the power to detect significant changes in the BALF sampling strategies.

Markers significantly elevated in the ventilated lung of patients with postoperative respiratory failure, including IL-1Ra, TARC, IL-7, IL-6, Eotaxin, IL-1α, and TGF-β2, were detected at higher levels in the ventilated lung of the complications group at baseline, even before OLV and lung surgery was initiated. The implication of this is that that there may be baseline preoperative inflammatory profiles for patients that are more likely to result in respiratory failure following lung surgery and, more importantly, that ***these can be detected preoperatively with a potential for therapies to prevent or reduce these risks***. IL-17D and IL-23 have a significant response to OLV with a four-fold and two-fold increase, respectively, in the ventilated lung of patients with complications. Both biomarkers appear to have a similar trend in both the complications and control patient groups, but the increase in IL-23 was more robust in the control group. The implication of this finding is that patients with complications may have a deficient IL-23 response to OLV. Thus, this may be a future drug target to help prevent/reduce post-operative respiratory complications. As a future direction, we can use an animal model with IL-23 blockade/knockout to assess this experimentally.

Previous lung surgery studies identified IL-6, IL-8, and IL-10 as systemic biomarkers that are elevated in patients with respiratory, cardiac, neurological, and renal complications at the time of wound closure and 24 h post procedure [26]. Our inclusion of lung fluid sampling at two perioperative timepoints, pre-OLV and post-OLV, suggests that systemic IL-6 levels may be elevated in these patients prior to OLV/surgery and further worsened by OLV as the increased systemic levels are reflected in the ventilated lung patterns of patients with respiratory complications. Thus, the implication of this is that there are groups of patients that are already “primed” for experiencing increased circulating inflammatory response to OLV; in other words, some patients may have a pre-existing risk for developing a harmful inflammatory response to OLV and lung surgery. These patients can potentially be targeted for anti-inflammatory medications pre-operatively or intra-operatively. This can be tested in future animal models in in vivo studies, as well as human clinical trials.

In our study, the systemic IL-10 concentration displayed the greatest magnitude in variability, an average of 13-fold increase in complications, when comparing the pre- to post-OLV arterial sampling, but we did not identify this dynamics as significant in our small population study. Thus, this suggests that IL-10 may be an important mediator of harmful inflammatory signals, but that larger samples are required to detect this signal.

***Ventilated lung biomarkers:*** Increasing levels of IL-6 in BALF and plasma sampling is a predominant cytokine response to OLV, and IL-1 receptor agonists have been detected in the BALFs following OLV [8]. IL-1Ra is an IL-1 receptor antagonist of IL-1β- and IL-1α-mediated inflammation [27,28]. Within the lung tissue, recruited interstitial macrophages and resident alveolar macrophages mediate anti-inflammatory and inflammatory signals, through IL-10, TNF-α, IL-6, and IL-1 and TGF-β signaling, respectively [29]. IL-1Ra has been reported to have anti-inflammatory properties and mediate the inflammatory response of infiltrating immune cells through the localized ratios of IL-1 to IL-1Ra proteins [28,30]. In our study, in patients with complications, IL-1β decreased in response to OLV from higher systemic levels. This complications group also had elevated levels of IL-1α in the ventilated lung pre-OLV when compared to control patients. Furthermore, IL-1α increased specifically in the ventilated lung of the complications group in response to OLV. A plethora of cytokines are able to stimulate IL-Ra production from monocytes in culture [31], including the IL-6 and TGF-β molecules detected in our study. IL-1RA-based therapies have demonstrated efficacy in reducing vascular permeability in VILI and SARS-CoV-2-stimulated lung injury animal models [32,33]. In a series of clinical studies, elevated systemic IL-1Ra levels have been associated with the early stages of non-small cell lung cancer [34], smoking status [35], VILI-induced lung damage in severe ARDS patients [36], and poor outcome in pediatric ARDS [37]. Elevated levels of two chemokines TARC and Eotaxin in the bronchoalveolar fluid of ventilated lungs is significant in patients with complications [38]. IL-7 has anti-apoptotic function in T-cell regulation. Although the role of IL-7 in ALI is unclear, elevated IL-7 has been associated with the most-severe cases of ARDS in COVID-19-infected patients [39]. Collectively, enhanced proinflammatory, immune cell recruitment and survival and imbalances in IL-1 dominant pro- and anti-inflammatory signaling characterized the ventilated lungs of OLV patients with postoperative respiratory failure in our current study. Thus, our findings in the context of the existing literature suggest that the IL-1 axis is a potential drug target for modulating the inflammatory response to OLV lung surgery and that existing ILRA drugs can be used to test this hypothesis in animal models.

***Operated lung biomarkers:*** A subset of innate lymphoid cells is functionally similar to Th17/Th22 immune cells, and the secretion of IL-22 by these cells is a suspected mediator of epithelial tissue repair [29]. In our work, IL-22 was elevated in the operated lungs of patients with complications pre-OLV, and this group was deficient for increasing concentrations observed in the control group. IL-17C is significantly elevated in the operated lungs of complications, while IL-21 is elevated in the operated lungs of controls. The ventilated lung responds to OLV with increasing concentrations of IL-23 and IL-17D, where the increase in IL-23 is more pronounced in the control group. Our results suggest that both the ventilated and operated lung are contributing to complications through a distinct IL-17/IL-22 response, and this is an under-investigated mechanism of inflammation specific to OLV.

***Systemic circulating biomarkers:*** Several proinflammatory molecules, including TNF-α, IFN-γ, and MCP-1, and the VEGF growth factor decrease in systemic levels in response to OLV. This decrease may reflect the acute response to injury sustained during OLV and the proximity of our systemic sampling to the OLV procedure. The recruitment of immune cells from circulation in an emergent immune response may account for the temporary drop in the levels of TNF-α typically observed in response to OLV [8]. Similarly, the VEGF ligand may be rapidly sequestered from circulation in a mechanotransduction response typical of VILI within the lung epithelia [40]. Consistent with the role of tissue trauma in acute lung injury [41], the levels of the soluble VEGF receptor, FLT-1, increased within both patient groups. Although all of the above biomarkers responded to the OLV procedure, none were associated with the complications or control group. Interestingly, both VEGF and FLT-1 haplotypes have been identified, which are associated with complications following lung surgery [42].

***Conclusions:*** This case–control study used unique sampling of local BALF and circulating biomarkers of inflammation and tissue injury prior to and just after OLV in the contact of lung surgery. This provided a unique assessment of the differences in localized and systemic markers of tissue injury and inflammation in those who developed respiratory failure after surgery versus matched controls who did not develop respiratory failure after surgery. This has never been previously reported, and thus, these are important hypothesis-generating findings that will require further focused investigation. Our findings demonstrated that there were unique biomarker changes that occur in the operated and ventilated lung, as well as in the circulating plasma compartment. Based on our assessment of the IL6 response, it appears that there may be groups of patients that are already “primed” for experiencing increased circulating inflammatory response to OLV; these patients can potentially be targeted for anti-inflammatory medications pre-operatively or intra-operatively. Our results suggest that both the ventilated and operated lung are contributing to complications through distinct IL-17/IL-22/IL-23 response, and this is an under-investigated mechanism of inflammation specific to OLV lung surgery. Our findings suggest that the IL-1 axis is a potential drug target for modulating the inflammatory response to OLV lung surgery and that existing ILRA drugs can be used to test this hypothesis in animal models. This can be tested in future animal models in in vivo studies, as well as human clinical trials. We established a large animal in vivo OLV lung surgery model to test these hypotheses further.

## 4. Materials and Methods

A case–control study nested within a prospective cohort study was performed. Consecutive patients undergoing lung resection were recruited from a single center regional thoracic surgical unit in Canada. From 17 December 2018 to 16 June 2021, all patients scheduled for lung surgery were approached for recruitment to a prospective cohort study. Patients were eligible for inclusion if they were deemed operable enough to be booked for lung surgery and if they were capable of consent. This study was approved by the regional Heath Research Ethics Board (HREB) HS22088 (H2018:334).

In the prospective cohort study, patients arrived in the operating room and underwent general anesthetic. Following intubation with an endotracheal tube (ETT), two-lung ventilation (TLV) was performed in the standard fashion. Before the initiation of OLV, bronchial alveolar lavage fluid (BALF) samples were collected via bronchoscope-guided sampling separately from each side of the lung (i.e., the side that will be ventilated and the side that will be operated on). An endotracheal tube (ETT) was used to perform the BALF collection by instilling and collecting sterile saline from the alveolar epithelium. The expelled and retrieved volume were recorded, as well as the sample qualities during the sampling processing. Arterial blood draws were sampled at this pre-OLV timepoint in 6 mL EDTA tubes. Then, OLV was initiated with the ventilation of the one ventilated lung while the surgical procedure (i.e., lung resection) was being performed on the operated contralateral lung. After the surgical procedure was completed, the operated lung could be re-inflated, and thus, OLV was stopped, then two-lung ventilation was resumed. At this post-OLV timepoint, the BALF samples were collected again via bronchoscope-guided sampling separately from each side of the lung (i.e., the side that was ventilated and the side that was operated on). Arterial blood draws were sampled again at this post-OLV timepoint in 6 mL EDTA tubes. Patients were then aroused from anesthesia, and the ETT was removed. Patients then underwent routine post-operative care. Post-operative complications were documented prospectively according to the type (pulmonary, pleural, cardiac, renal, gastrointestinal, neurological, and wound-associated) and the grade of complications using the standardized and validated Ottawa Thoracic Morbidity and Mortality classification system [43]. The occurrence of these complications was doubly adjudicated and verified (first by the Attending Thoracic Surgeon, then separately by the Director of Thoracic Surgery Quality Assurance).

A total of 209 patients underwent the prospective cohort study. Prospective collection was performed on preoperative variables including preoperative diagnosis, the occurrence of cancer with biopsy pathology reports, the age at the time of surgery, biological sex, the Body Mass Index (BMI), pre-existing chronic pulmonary disease including interstitial lung disease (ILD), chronic obstructive pulmonary disease (COPD), asthma, emphysema, obstructive sleep apnea (OSA), pulmonary hypertension (PH), pleural disease, and smoking status in pack-years, and patient comorbidities were documented by the attending physician. Documented also were the baseline pulmonary function tests (PFTs) including the baseline O_2_ saturation, the forced expiratory volume in one second (FEV_1_), the forced vital capacity (FVC), and the diffusing capacity of the lungs for carbon monoxide (DLCO). %FEV1/FVC was calculated as an indicator of normal, obstructive, or restricted airways (restrictive). The surgical procedure was recorded including the type of surgical procedure (open vs. VATS), the extent of lung resection (wedge resection vs. lobectomy), and the location of the operative procedure (right vs. left lung and upper, middle, or lower lobes). The procedure date, start time, duration, and discharge date were documented. Intraoperative mechanical ventilation and postoperative extubation recovery were recorded including the duration of two-lung ventilation (TLV) and one-lung ventilation (OLV). Other interventions recorded included steroid use and dose, as well the use of regional nerve analgesia (i.e., paravertebral, intercostal, or epidural).

In order to perform a nested case–control study, we focused on patients who developed post-operative respiratory failure, and these were termed the complications. Post-operative respiratory failure was defined a priori as: the need for continued mechanical ventilation greater than 48 h immediately after surgery ***or*** the need for any new mechanical ventilation beyond the first post-operative day or new requirement for home oxygen use. We screened all 209 patients from the prospective cohort study to identify these cases of complications. Six patients (IDs 9, 44, 47, 54, 76, and 183; Table 3) who had respiratory failure were identified. The causality of respiratory failure was verified to remove respiratory failure cases that were likely to be caused by post-operative insults rather than intra-operative insults (i.e., one patient with respiratory failure was excluded because he/she only developed respiratory failure after suffering massive aspiration of food and emesis into his/her lungs on the second post-operative day). To find matched controls for these complications, we screened for patients (from the 209 patients in the prospective cohort study) that did not experience respiratory failure and that were good matches for the complications cases. Matching variables were selected based on important pre-operative/operative variables known to be predictors of developing post-operative respiratory complications/failure. These include a greater extent of lung removed and worse preoperative lung function (i.e., lower FEV1 or DLCO).

The variables associated with increased postoperative respiratory complications in the prospective cohort study included sex (five of six patients were males), the extent of lung and thoracic tissue removal (five of five patients with Grade 4 or 5 respiratory complications received lobectomies), and low calculated postoperative DLCO values (four of five patients had values below 60%). Thus, the complications patient cases were matched with five control patients that were similar in clinical variables and respiratory function (IDs 64, 66, 145, 146, and 182; Table 3. The highest priority for matching was to ensure a match on surgical type (i.e., wedge resections vs. lobectomies, as well as location within the organ including the upper and lower lobes), as well as minimizing the difference between the DLCO values for each pair of complication and control. Patient matches were further optimized to minimize mismatches based on sex and age and the FEV_1_, and %FEV_1_/FVC values.

Arterial blood and BALF samples were processed to determine the systemic and localized concentration of 55 biomarkers for two surgery timepoints: pre-OLV and post-OLV. The lung subject to surgical procedure and that to OLV were distinguished and defined as the operated and ventilated lung, respectively. Samples were stored on ice and processed immediately following the surgery. The blood samples were centrifuged at 4 °C for 5000 rpm for 10 min in a Sorval ST/6R centrifuge. BALF samples were inverted and centrifuged at 4 °C 3500 rpm for 5 min. The serum and BALF supernatants were aliquoted into a minimum of six 500 uL aliquots and stored at −80 °C. Patient sample aliquots were thawed on ice and briefly centrifuged at 4 °C to remove insoluble particulates prior to sample analysis. The MesoScaleDiscovery multiplex immunoassays were analyzed on a MESO QuickPlex SQ 120 Model 1300 plate reader with the Discovery Workbench 4.0.12 software package. The following V-plex and U-plex kits were used according to the manufacturer protocols. **Proinflammatory Panel 1** included interferon (IFN)-γ, interleukin (IL)-1β, IL-2, IL-4, IL-6, IL-8, IL-10, IL-12 (p70), IL-13, and tumor necrosis factor (TNF)-α; **Cytokine Panel 1** included granulocyte macrophage colony-stimulating factor (GM-CSF), IL-1α, IL-5, IL-7, IL-12/IL-23 (p40), IL-15, IL-16, IL-17A (to verify lot for comparison to TH17), TNF-β, and vascular endothelial growth factor (VEGF)-A; **Chemokine Panel 1** included Eotaxin, Eotaxin-3, IL-8 (HA), interferon γ-induced protein (IP)-10, monocyte chemoattractant protein (MCP)-1, MCP-4, mediator of DNA damage checkpoint protein (MDC), macrophage inflammatory protein (MIP)-1α, MIP-1β, thymus and activation regulated chemokine (TARC); **Cytokine Panel 2** included IL-17A/F, IL-17B, IL-17C, IL-17D, IL-1RA, IL-3, IL-9, and thymic stromal lymphopoietin (TSLP) K15084 (Lot No. K0081180), **TH17 Panel 1** included IL-17AGenB, IL-21, IL-22, IL-23, IL-27, IL-31, and MIP-3α; **Angiogenesis Panel 1** included VEGF-A, VEGF-C, VEGF-D, tyrosine kinase with immunoglobulin and EGF factor homology domains (Tie)-2, Fms-related receptor tyrosine kinase (Flt)-1, placenta growth factor (PIGF), basic fibroblast growth factor (bFGF); the U-plex TGF-β panel included TGF-β1, TGF-β2, and TGF-β3. For each 96-well assay, standards were run in duplicate and unknown sample positions were randomized. Samples were run as single reads or randomly selected duplicates. The average concentration for each protein was calculated using the Workbench 4.0 software standard curve algorithm. Sample reads below the level of detection (LOD) score for each biomarker were assigned the value of the lowest level of detection divided by ten for bioinformatic analysis.

Of the 55 total analytes, three (IL-17B, IL-9i, IL-3) were not included in the analysis because a majority of observations were below the level of quantification (LOQ). Another four (IL-8HA, IL-17A, IL-17A_Fi, VEGF) were not included because they were analyte duplicates. Samples with duplicate measure were calculated as the mean for the analysis. Fold-change and ANOVA analysis for each sample type were analyzed in R-programming. For each cytokine, the means of the pre- and post-OLV values were taken for all patients according to their identified group type (i.e., complications or control).

Then, the fold-change was calculated by dividing the two means (i.e., the mean of post-OLV samples/the mean of pre-OLV samples). For graphical visualization, the log_2_ of the fold-change was calculated and plotted. An infinite fold-change was indicated, where the pre-OLV mean was 0 (i.e., below the LOQ value) or a 0 fold-change because both the pre- and post-OLV mean values were 0 (i.e., not detected). In the case of infinity, the fold-change was assigned an arbitrary fold-change at the edge of the plot so it could be shown. A repeated measures ANOVA was performed on the samples, and any test with a *p*-value of less than 0.05 was considered significant. For each sample type (i.e., ventilated lung BALF, operated lung BALF, and arterial plasma), the data were grouped by analyte. The ANOVA RM test had the formula: anova_test(values∼type×sample+Error(id/sample)), where *values* is equal to the cytokine abundances for each patient, *type* indicates if the patient belongs to the complications or control group type, and *sample* indicates if the sample was collected pre- or post-OLV/surgery. For all the significant analytes, the raw data were plotted, and a linear model was created to test the normality assumption. The *p*-values for the significant relationships are annotated within the table and on the dot plots with the following annotations: p_1_ patient type effect (PT), p_2_ sample time effect (ST), and p_3_ patient type:sample time full effect (PT:ST). The linear model had the formula: $lm\left(values\sim type\times sample\right)$ · *y*. Four tests were performed on the created linear model including the residual QQ plot, the histogram of the residuals, the correlation between the observed residuals, and the expected residuals under normality, as well as a test for detecting the violation of the normality assumption. The R-packages (v4.0.3) used were: readxl, tidyverse, rstatix, flextable, and olsrr.

## Figures and Tables

**Table 1:** Biomarkers detected in the bronchial alveolar lavage fluids with total concentration change differentials greater than 2-fold when the change in mean concentrations pre-OLV to post-OLV were compared between the complications and control groups. Patient type (PT), sample time (ST), patient type:sample type (PT:ST).

Sample Location	Fold-Change (log2)			ANOVA RM F-Score (*p*-Value) α * ≤ 0.05 α ** ≤ 0.001		
Biomarker	Complication Mean	Control Mean	Δ	PT (p_1_)	ST (p_2_)	PT:ST (p_3_)
***Ventilated Lung***						
Tie-2	−∞	1.93	+∞	1.305 (0.283)	1.353 (0.275)	2.410 (0.155)
TNF-α	−0.50	3.48	3.98	1.608 (0.245)	2.011 (0.199)	2.468 (0.160)
IL-12 (p70)	−0.60	2.73	3.33	2.813 (0.137)	2.012 (0.199)	2.890 (0.133)
MIP-1α	0.57	3.26	2.69	1.891 (0.202)	1.925 (0.199)	1.733 (0.221)
INF-γ	0.17	2.54	2.37	1.566 (0.251)	1.265 (0.298)	1.115 (0.326)
TGF-β3	0	2.16	2.16	2.053 (0.195)	1.972 (0.203)	1.972 (0.203)
MIP-1β	1.30	3.42	2.12	1.397 (0.267)	1.603 (0.237)	1.307 (0.282)
IL-17D	3.42	5.42	2.00	1.430 (0.262)	6.699 (0.029) *	1.590 (0.239)
IL-10	0.55	2.45	1.90	2.084 (0.192)	3.159 (0.119)	2.068 (0.194)
IL-8	−0.05	1.34	1.39	1.247 (0.301)	1.975 (0.203)	2.159 (0.185)
IL-13	−0.02	1.34	1.36	0.861 (0.384)	1.469 (0.265)	1.518 (0.258)
IL-4	0.12	1.39	1.27	3.474 (0.105)	1.221 (0.306)	1.072 (0.335)
IL-1β	0.55	1.79	1.24	3.354 (0.110)	4.803 (0.065)	2.929 (0.131)
IL-23	2.85	4	1.15	7.475 (0.023) *	8.280 (0.018) *	3.685 (0.087)
IL-2	−0.78	0.32	1.10	1.355 (0.283)	0.023 (0.885)	0.661 (0.443)
IL-16	0.70	1.76	1.06	2.289 (0.169)	1.932 (0.202)	1.480 (0.258)
TSLP	∞	3.00	−∞	1.153 (0.311)	4.891 (0.054) *	0.571 (0.469)
bFGF	2.48	0.80	−1.68	0.649 (0.441)	4.960 (0.053) *	0.001 (0.979)
IL-21	0.80	−0.74	−1.54	1.317 (0.281)	0.174 (0.686)	3.620 (0.090)
Flt-1	1.5	0.08	−1.42	0.392 (0.547)	1.107 (0.320)	0.787 (0.398)
IL-1Ra	1.42	0.11	−1.31	11.506 (0.008) **	0.542 (0.481)	0.076 (0.789)
IL-6	1.41	0.11	−1.30	6.480 (0.038) *	0.175 (0.689)	0.023 (0.883)
***Operated Lung***						
Tie-2	−∞	0.01	∞	0.601 (0.458)	0.372 (0.557)	0.393 (0.546)
VEGF-C	−∞	0.03	∞	2.342 (0.160)	0.040 (0.847)	0.061 (0.811)
MIP-3α	1.83	4.1	2.27	0.881 (0.372)	0.881 (0.372)	0.775 (0.402)
VEGF-D	0.91	2.24	1.33	2.468 (0.151)	4.582 (0.061)	2.710 (0.134)
TGF-β3	∞	−∞	−∞	0.215 (0.657)	0.215 (0.657)	1.450 (0.268)
TSLP	∞	1.82	−∞	0.301 (0.597)	3.249 (0.105)	0.000 (0.992)
bFGF	3.1	0.44	−2.66	0.668 (0.435)	4.113 (0.073)	0.329 (0.580)
INF-γ	0.29	−2.36	−2.65	4.783 (0.065)	0.157 (0.704)	0.780 (0.406)
PlGF	1.82	−0.69	−2.51	3.201 (0.107)	0.006 (0.939)	0.967 (0.351)
IL-6	2.49	0.02	−2.47	1.617 (0.244)	1.165 (0.316)	1.089 (0.331)
TGF-β2	0.46	−1.67	−2.13	0.100 (0.761)	1.246 (0.301)	3.641 (0.098)
IL-1β	0.5	−1.37	−1.87	0.659 (0.444)	0.291 (0.606)	0.729 (0.421)
IL-17C	1.21	−0.5	−1.71	8.769 (0.016) *	0.000 (0.983)	1.680 (0.227)
IL-1α	1.06	−0.52	−1.58	1.890 (0.206)	0.026 (0.877)	0.205 (0.663)
IL-22	1.24	−0.21	−1.45	0.268 (0.617)	3.558 (0.092)	6.630 (0.030) *
IL-2	0.62	−0.83	−1.45	0.321 (0.589)	0.000 (0.986)	1.271 (0.297)
IL-8	0.59	−0.66	−1.25	0.331 (0.583)	0.004 (0.949)	1.406 (0.274)
IL-10	1.72	0.51	−1.21	5.130 (0.058)	2.913 (0.132)	1.541 (0.254)
IL-5	1.74	0.66	−1.08	0.456 (0.519)	0.871 (0.378)	0.045 (0.838)

**Table 2:** Biomarkers detected in the arterial plasma sampling with total concentration change differentials greater than 2-fold when the change in mean concentrations pre-OLV to post-OLV were compared between the complications and control groups. Patient type (PT), sample time (ST), patient type:sample type (PT:ST).

	Fold-Change (log2)			ANOVA RM F-Score (*p*-Value) α * ≤ 0.05 α ** ≤ 0.001		
Biomarker	Complication Mean	Control Mean	Δ	PT	ST	PT:ST
IL-10	3.43	7.13	3.70	2.279 (0.175)	3.111 (0.121)	2.275 (0.175)
IL-17D	−0.04	2.35	2.39	0.165 (0.694)	0.985 (0.347)	1.038 (0.335)
Eotaxin-3	−0.44	0.57	1.01	1.340 (0.277)	0.459 (0.515)	1.603 (0.237)
VEGF-C	2.78	−0.34	−3.12	1.408 (0.266)	1.140 (0.313)	1.327 (0.279)
GM-CSF	1.7	−0.33	−2.03	0.009 (0.926)	0.590 (0.465)	1.562 (0.247)
IL-1Ra	1.79	0	−1.79	0.046 (0.834)	1.733 (0.221)	1.714 (0.223)
IL-6	2.45	0.73	−1.72	2.385 (0.166)	6.654 (0.036) *	0.141 (0.718)
IL-1β	−0.07	−1.47	−1.4	1.218 (0.306)	15.207 (0.006) **	13.379 (0.008) **
IFN-γ	−0.81	−2.1	−1.29	2.637 (0.148)	12.899 (0.009) **	0.126 (0.733)

**Table 3:** Surgical variables and baseline respiratory assessments for study lung surgery patients.

Patient ID	Surgery Type	OLV ^1^	TLV ^1^	Sex	Age	BMI	DLCO	FEV_1_	%FEV_1_/FVC	Smoking Status (PY)
9	VATS RLL-WR	1:06	0:38	M	77	33.8	61	114	113	125
44	VATS RLL-L	3:11	1:12	M	78	23.9	80	70	58	30.5
47	VATS RUL-L ^2^	5:35	1:16	M	59	29.7	46	73	83	27
54	VATS RUL-L ^3^	2:55	1:13	F	69	20.6	47	97	79	33
64	VATS RUL-L	1:49	0:43	F	74	21.1	53	88	100	16.8
66	VATS LLL-L	3:52	0:41	M	78	27.4	82	70	77	20
76	VATS LUL-L	4:14	1:19	M	51	44.6	58	71	102	95
145	VATS LLL-L	3:17	1:08	F	72	21.8	47	58	77	12.5
146	VATS RUL-L	1:32 ^4^	NA	F	72	30.4	56	69	81	18
182	VATS LLL-L	2:58	1:05	F	80	21.6	51	106	72	40
183	VATS RUL-L	4:16	0:55	M	66	29.2	52	74	80	40

Abbreviations: VATS: video-assisted thoracic surgery; R: right; L: left, UL upper lobe; LL: lower Lobe; L: lobectomy; WR: wedge resection; OLV: one-lung ventilation; TLV: two-lung ventilation; M: male; F: female; DLCO: diffusing lung capacity for carbon monoxide; FEV_1_: forced expiratory volume in 1 s; FVC: forced vital capacity; CPD: chronic pulmonary disease; ILD: interstitial lung disease; COPD: chronic obstructive lung disease; Mix CPD: mixed-presentation CPD; PY: pack-years; BMI: Body Mass Index (kg/m); NA: not available due to the COVID-19 protocol. ^1^ Time in hours:minutes; ^2^ mini-thoracotomy; ^3^ middle lobe and lower lobe pneumopexies; ^4^ estimate based on BALF time.

## Data Availability

The data are available upon request due to privacy restrictions.

## References

[1] Bray F., Ferlay J., Soerjomataram I., Siegel R.L., Torre L.A., Jemal A. (2018). 394 CA: A Cancer Journal for Clinicians Global Cancer Statistics 2018: GLOBOCAN Estimates of Incidence and Mortality Worldwide for 36 Cancers in 185 Countries. CA Cancer J. Clin..

[2] Lang-Lazdunski L. (2013). Surgery for Nonsmall Cell Lung Cancer. Eur. Respir. Rev..

[3] Brassard C.L., Lohser J., Donati F., Bussiè J.S. (2014). Step-by-Step Clinical Management of One-Lung Ventilation: Continuing Professional Development. Can. J. Anesth..

[4] Grichnik K.P., Clark J.A. (2005). Pathophysiology and Management of One-Lung Ventilation. Thorac. Surg. Clin..

[5] Della Rocca G., Coccia C. (2013). Acute Lung Injury in Thoracic Surgery. Curr. Opin. Anesthesiol..

[6] Della Rocca G., Coccia C. (2011). Ventilatory Management of One-Lung Ventilation. Minerva Anestesiol..

[7] Gama de Abreu M., Heintz M., Heller A., Széchényi R., Albrecht D.M., Koch T. (2003). One-Lung Ventilation with High Tidal Volumes and Zero Positive End-Expiratory Pressure Is Injurious in the Isolated Rabbit Lung Model. Anesth. Analg..

[8] Bruinooge A.J.G., Mao R., Gottschalk T.H., Srinathan S.K., Buduhan G., Tan L., Halayko A.J., Kidane B. (2022). Identifying Biomarkers of Ventilator Induced Lung Injury during One-Lung Ventilation Surgery: A Scoping Review. J. Thorac. Dis..

[9] Peel J.K., Funk D.J., Slinger P., Srinathan S., Kidane B. (2020). Positive End-Expiratory Pressure and Recruitment Maneuvers during One-Lung Ventilation: A Systematic Review and Meta-Analysis. J. Thorac. Cardiovasc. Surg..

[10] Peel J.K., Funk D.J., Slinger P., Srinathan S., Kidane B. (2022). Tidal Volume during 1-Lung Ventilation: A Systematic Review and Meta-Analysis. J. Thorac. Cardiovasc. Surg..

[11] Kidane B., Choi S., Fortin D., O’hare T., Nicolaou G., Badner N.H., Inculet R.I., Slinger P., Malthaner R.A. (2018). Use of Lung-Protective Strategies during One-Lung Ventilation Surgery: A Multi-Institutional Survey. Ann. Transl. Med..

[12] Dulu A., Pastores S.M., Park B., Riedel E., Rusch V., Halpern N.A. (2006). Prevalence and Mortality of Acute Lung Injury and ARDS after Lung Resection. Chest.

[13] Alam N., Park B.J., Wilton A., Seshan V.E., Bains M.S., Downey R.J., Flores R.M., Rizk N., Rusch V.W., Amar D. (2007). Incidence and Risk Factors for Lung Injury After Lung Cancer Resection. Ann. Thorac. Surg..

[14] Ruffni E., Parola A., Papalia E., Filosso P.L., Mancuso M., Oliaro A., Actis-Dato G., Maggi G. (2001). Frequency and Mortality of Acute Lung Injury and Acute Respiratory Distress Syndrome after Pulmonary Resection for Bronchogenic Carcinoma Q. Eur. J. Cardio-Thorac. Surg..

[15] Licker M., de Perrot M., Spiliopoulos A., Robert J., Diaper J., Chevalley C., Tschopp J.-M. (2003). Risk Factors for Acute Lung Injury after Thoracic Surgery for Lung Cancer. Anesth. Analg..

[16] Eichenbaum K.D., Neustein S.M. (2010). Acute Lung Injury After Thoracic Surgery. J. Cardiothorac. Vasc. Anesth..

[17] Relja B., Yang B., Bundkirchen K., Xu B., Köhler K., Neunaber C. (2020). Different Experimental Multiple Trauma Models Induce Comparable Inflammation and Organ Injury. Sci. Rep..

[18] Weckbach S., Hohmann C., Braumueller S., Denk S., Klohs B., Stahel P.F., Gebhard F., Huber-Lang M.S., Perl M. (2013). Inflammatory and Apoptotic Alterations in Serum and Injured Tissue after Experimental Polytrauma in Mice: Distinct Early Response Compared with Single Trauma or “Double-Hit” Injury. J. Trauma Acute Care Surg..

[19] Brunelli A., Cicconi S., Decaluwe H., Szanto Z., Falcoz P.E. (2020). Parsimonious Eurolung Risk Models to Predict Cardiopulmonary Morbidity and Mortality Following Anatomic Lung Resections: An Updated Analysis from the European Society of Thoracic Surgeons Database. Eur. J. Cardio-Thorac. Surg..

[20] Brunelli A., Salati M., Rocco G., Varela G., Van Raemdonck D., Decaluwe H., Falcoz P.E. (2017). European Risk Models for Morbidity (EuroLung1) and Mortality (EuroLung2) to Predict Outcome Following Anatomic Lung Resections: An Analysis from the European Society of Thoracic Surgeons Database. Eur. J. Cardio-Thorac. Surg..

[21] Kozower B.D., Sheng S., O’Brien S.M., Liptay M.J., Lau C.L., Jones D.R., Shahian D.M., Wright C.D. (2010). STS Database Risk Models: Predictors of Mortality and Major Morbidity for Lung Cancer Resection. Ann. Thorac. Surg..

[22] Fernandez F.G., Kosinski A.S., Burfeind W., Park B., DeCamp M.M., Seder C., Marshall B., Magee M.J., Wright C.D., Kozower B.D. (2016). The Society of Thoracic Surgeons Lung Cancer Resection Risk Model: Higher Quality Data and Superior Outcomes. Ann. Thorac. Surg..

[23] Ferguson M.K., Dignam J.J., Siddique J., Vigneswaran W.T., Celauro A.D., Ferguson M.K. (2012). Diffusing Capacity Predicts Long-Term Survival after Lung Resection for Cancer. Eur. J. Cardio-Thorac. Surg..

[24] Müller-Redetzky H.C., Felten M., Hellwig K., Wienhold S.-M., Naujoks J., Opitz B., Kershaw O., Gruber A.D., Suttorp N., Witzenrath M. (2015). Increasing the Inspiratory Time and I:E Ratio during Mechanical Ventilation Aggravates Ventilator-Induced Lung Injury in Mice. Crit. Care.

[25] Han B. (2005). Ventilator-Induced Lung Injury: Role of Protein-Protein Interaction in Mechanosensation. Proc. Am. Thorac. Soc..

[26] Kaufmann K.B., Heinrich S., Felix Staehle H., Bogatyreva L., Buerkle H., Goebel U. (2018). Perioperative Cytokine Profile during Lung Surgery Predicts Patients at Risk for Postoperative Complications-A Prospective, Clinical Study. PLoS ONE.

[27] Arend W.P., Malyak M., Guthridge C.J., Gabay C. (1998). Interleukin-1 Receptor Antagonist: Role in Biology. Annu. Rev. Immunol..

[28] Arend W.P. (2002). The Balance between IL-1 and IL-1Ra in Disease. Cytokine Growth Factor Rev..

[29] Ardain A., Marakalala M.J., Leslie A. (2020). Tissue-Resident Innate Immunity in the Lung. Immunology.

[30] Marriott H.M., Gascoyne K.A., Gowda R., Geary I., Nicklin M.J.H., Iannelli F., Pozzi G., Mitchell T.J., Whyte M.K.B., Sabroe I. (2012). Interleukin-1β Regulates CXCL8 Release and Influences Disease Outcome in Response to *Streptococcus Pneumoniae*, Defining Intercellular Cooperation between Pulmonary Epithelial Cells and Macrophages. Infect. Immun..

[31] Ravandi A., Leibundgut G., Hung M.-Y., Patel M., Hutchins P.M., Murphy R.C., Prasad A., Mahmud E., Miller Y.I., Dennis E.A. (2014). Release and Capture of Bioactive Oxidized Phospholipids and Oxidized Cholesteryl Esters during Percutaneous Coronary and Peripheral Arterial Interventions in Humans. J. Am. Coll. Cardiol..

[32] Xiong S., Zhang L., Richner J.M., Class J., Rehman J., Malik A.B. (2021). Interleukin-1RA Mitigates SARS-CoV-2-Induced Inflammatory Lung Vascular Leakage and Mortality in Humanized K18-HACE-2 Mice. Arterioscler. Thromb. Vasc. Biol..

[33] Frank J.A., Pittet J.-F., Wray C., Matthay M.A. (2008). Protection from Experimental Ventilator-Induced Acute Lung Injury by IL-1 Receptor Blockade. Thorax.

[34] Farlow E.C., Vercillo M.S., Coon J.S., Basu S., Kim A.W., Faber L.P., Warren W.H., Bonomi P., Liptay M.J., Borgia J.A. (2010). A Multi-Analyte Serum Test for the Detection of Non-Small Cell Lung Cancer. Br. J. Cancer.

[35] Lugg S.T., Alridge K.A., Howells P.A., Parekh D., Scott A., Mahida R.Y., Park D., Tucker O., Gao F., Perkins G.D. (2019). Dysregulated Alveolar Function and Complications in Smokers Following Oesophagectomy. ERJ Open Res..

[36] Del Sorbo L., Goffi A., Tomlinson G., Pettenuzzo T., Facchin F., Vendramin A., Goligher E.C., Cypel M., Slutsky A.S., Keshavjee S. (2020). Effect of Driving Pressure Change During Extracorporeal Membrane Oxygenation in Adults With Acute Respiratory Distress Syndrome: A Randomized Crossover Physiologic Study. Crit. Care Med..

[37] Dahmer M.K., Quasney M.W., Sapru A., Gildengorin G., Curley M.A.Q., Matthay M.A., Flori H. (2018). Interleukin-1 Receptor Antagonist Is Associated With Pediatric Acute Respiratory Distress Syndrome and Worse Outcomes in Children With Acute Respiratory Failure. Pediatr. Crit. Care Med..

[38] Manicone A.M. (2009). Role of the Pulmonary Epithelium and Inflammatory Signals in Acute Lung Injury. Expert Rev. Clin. Immunol..

[39] Kalinina O., Golovkin A., Zaikova E., Aquino A., Bezrukikh V., Melnik O., Vasilieva E., Karonova T., Kudryavtsev I., Shlyakhto E. (2022). Cytokine Storm Signature in Patients with Moderate and Severe COVID-19. Int. J. Mol. Sci..

[40] Tian Y., Gawlak G., O’Donnell J.J., Birukova A.A., Birukov K.G. (2016). Activation of Vascular Endothelial Growth Factor (VEGF) Receptor 2 Mediates Endothelial Permeability Caused by Cyclic Stretch. J. Biol. Chem..

[41] Lassus P., Turanlahti M., Heikkilä P., Andersson L.C., Nupponen I., Sarnesto A., Andersson S. (2001). Pulmonary Vascular Endothelial Growth Factor and Flt-1 in Fetuses, in Acute and Chronic Lung Disease, and in Persistent Pulmonary Hypertension of the Newborn. Am. J. Respir. Crit. Care Med..

[42] Kim J.Y., Hildebrandt M.A.T., Pu X., Ye Y., Correa A.M., Vaporciyan A.A., Wu X., Roth J.A. (2012). Variations in the Vascular Endothelial Growth Factor Pathway Predict Pulmonary Complications. Ann. Thorac. Surg..

[43] Seely A.J., Anstee C., Gilbert S., Maziak D.E., Moffat-Bruce S., Sundaresan S., Villeneuve P.J. The Ottawa Thoracic Morbidity & Mortality System Classifying Thoracic Surgical Complications. https://ottawatmm.org.

